# New Drugs, Appliances, and Things Medical

**Published:** 1890-11-29

**Authors:** 


### NEW DRUGS, APPLIANCES, AND THINGS MEDICAL.

[All preparations, appliances, novelties, etc., of which a notice is desired, should be sent for The Editor, to care of The Manager, 140, Strand, London, W.C.]

### TEA, COFFEE, OR COCOA?

Recently we commented on the value of cocoa as a food and gave the comparison between it and the most typical form of animal food, namely, beefsteak. In this issue we shall say a few words as to why it is in part supplanting tea, we do not add coffee, for the general public has never drunk *pure* good coffee in the same way as it has tea, chiefly we fancy because the price of coffee is considerably higher. A pound of tea will go much farther than a pound of pure coffee at the same price. It will be noted that we say pure coffee, the ordinary fluid served out under that name too often has a large admixture of chicory and other diluents and adulterants in it. The objections to coffee are not many, but it has the popular attribute of being "bilious." By that we suppose is meant, that it repeats for some time after being swallowed, and certainly it sometimes causes a peculiar heart action. In its favour are the facts that it gives to many the desired stimulation either for work, mental or bodily, or for digestion, and also that it contains but little active tannin. But let us take tea. We suppose that since the tax has been practically reduced to nil, that of all drinks tea heads the list. What are the objections to tea? Setting aside particular idiosyncrasies and the over indulgence in the cup that cheers and does stimulate, the objections are mainly those caused by the tannin derivatives present. It is a well known fact that anything of an albuminous nature takes from two to four or even five times as long to digest in the presence of these derivatives as when they are absent. Moreover, it is probable that a large proportion of the albuminous tannates are simply wasted by being excreted before they can be digested. Then the process of digestion of albumens in the presence of tannin is an unpleasant one; there is often pain, heartburn, and flatulence. These evils were not so much noticed in the old days of pure China teas. Now the public taste is so vitiated that good China teas are not considered strong enough, and the strong and highly flavoured Indian teas have taken their place. It is no unusual thing for a lady making afternoon calls to drink three or four cups of tea in about two hours. Need we wonder that there is no appetite for dinner, and that vomiting and nausea are not unfrequently met with as the immediate effects of the tea bibbing which goes on at practically every lady's "at home." Now, we hear some one say, "Oh that's all very well, these things are the exception, not the rule." If you ask any general practitioner in active work whether he has seen anything of the sort, he will answer "repeatedly."

1. The total food value of cocoa is largely above that of coffee or tea. It is both food and drink.

Dr. Pavy, in his work on "Foods," says of cocoa: "Containing, as pure cocoa does, twice as much nitrogenous matter, and twenty-five times as much fatty matter as wheaten flour, with a notable quantity of starch and an agreeable aroma to tempt the palate, it cannot be otherwise than a valuable alimentary material."

Again, Dr. Parkes, in his "Hygiene," says of cocoa: "The large quantity of fat and albumenoid (flesh-forming) substance makes it a very nourishing article of diet; and it is therefore useful in weak state of the system and for health, even under circumstances of great exertion. *It has even been compared to milk.*"

2. Its stimulating power, due to the presence of the alkaloid theobromine, is equal to that of tea or coffee.

3. The absence of the tannin and its compounds which exist in tea, and to a certain extent in coffee, enhances its value.

Tea contains, according to König (in A. W. Blyth's work on Foods) 12.36 per cent. of tannin. Coffee also contains it, but it is in a peculiar compound form, quite different in its behaviour to the free tannin of tea. Cocoa is practically free from tannin.

During the last year or so we have published notices of the Cocoa preparations of several firms. Among the oldest of these, we believe, is that of Van Houten. We have lately investigated their method of manufacture. So long ago as sixty-two years they were granted a patent in Holland for the manufacture of cocoa, and we believe they were the first to make cocoa more digestible by the expression of part of its fats (cocoa butter). In making cocoa from raw material, a number of processes have to be passed through. Van Houtens have invented one by which the nitrogenous basis has been increased in solubility by 50 per cent. This must, of course, greatly lessen the work of digestion. The firm, on our enquiry, has intimated the nature of their process, and we quite understand why it is their cocoa is so soluble. We consider that not only is Van Houten's cocoa particularly suited for quick and easy preparation for the general public, but the gouty and dyspeptic will find it an article of diet of great value. In proof that Van Houtens are among the pioneers of improved cocoa making, we may mention that their claim to be in the van of first-class cocoa makers has been acknowledged by the exhibition judges of many countries, who have awarded them no less than thirty-six medals and diplomas in the last seven years.

### "CHERRY BLOSSOM" SOAP.

Messrs. John Gosnell and Co., Upper Thames Street, have forwarded to us a sample of their "Cherry Blossom" Soap, No. 585. Our investigation of it bears out in full Messrs. Allen and Hanburys' published analysis. It is a clean looking transparent soap, scented with the well-known Cherry Blossom Perfume. There is no excess of alkali present, and the fatty acids are well combined with the alkali (soda). It is decidedly a good article, which suits delicate skins, and ladies and children especially will be benefited by its use.

